# Single-Actuator-Based Lower-Limb Soft Exoskeleton for Preswing Gait Assistance

**DOI:** 10.1155/2020/5927657

**Published:** 2020-07-09

**Authors:** Ming-Hwa Hsieh, Yin Hsuan Huang, Chia-Lun Chao, Chien-Hao Liu, Wei-Li Hsu, Wen-Pin Shih

**Affiliations:** ^1^Department of Mechanical Engineering, National Taiwan University, Taipei 10617, Taiwan; ^2^The School and Graduate Institute of Physical Therapy College of Medicine, National Taiwan University, Taipei 10617, Taiwan

## Abstract

In this research, we proposed a lower-limb soft exoskeleton for providing assistive forces to patients with muscle weakness during the preswing phase of a gait cycle. Whereas conventional soft exoskeletons employ two motors to assist each leg individually, we designed a single motor for actuation. Our design assists hip flexion for light weights and prevents some slip problems that can arise from rotary motors. The actuation mechanism was based on a pulley system that converted the power supplied by the single motor into linear reciprocating motions of a slider. When the single motor rotated, the slider moved linearly, first in one direction and then in the opposite direction. The slider pulled knee braces through cables with an assistive force of 100 N. The actuation was triggered when the system detected that the backward swing of the wearer's thigh had ended. A prototype was designed, fabricated, and examined with 7 subjects (average age, 24). Subjects were measured while they wore our exoskeleton in power-off and power-on modes. Comparisons proved that wearing the exoskeleton caused a negligible deviation of gait, and that the soft exoskeleton could reduce metabolic cost during walking. The research results are expected to be beneficial for lightweight soft exoskeletons and integration with exosuits that provide assistive forces through the wearer's entire gait.

## 1. Introduction

Robotic rigid exoskeletons are commonly used for various applications, including action assistance, augmentation, and rehabilitation. Rehabilitation-based exoskeletons are usually mounted on stationary facilities such as treadmills; patients with stroke or injuries wear these exoskeletons for gait retraining or rehabilitation [1–5]. Various portable rigid exoskeletons have been developed; impaired patients wear these to regain functional abilities. When paralyzed patients wear these exoskeletons, they can walk on their feet again [6]. Even if some elderly patients lack muscle strength, they can wear exoskeletons to regain their grabbing and gesturing abilities [7]. In addition, gear-based portable rigid exoskeletons can enhance the strength of healthy wearers for conducting heavy-weight tasks, such as carrying loads [8] and heavy lifting [9, 10], or to conduct long-term activities that require stamina, such as walking [11] and driving [12]. Recently, lower-limb exoskeletons have attracted considerable attention for providing walking assistance or load-carrying capability through because they can shift the load from the wearer and pass the load to the ground [13]. However, a disadvantage of rigid exoskeletons is that they are heavy and thus require undesired metabolic expenditures [14]; additionally, they usually impose kinematic constraints when the wearer tries to walk [15]. Few rigid exoskeletons can achieve metabolic reduction for problems such as angle assistance [16], regardless of whether they are worn for tethered or stationary activities [17].

Soft exoskeletons composed of artificial muscles and cable-based actuation mechanisms have become very popular in the early twenty-first century because they are lightweight and comfortable. Traditional air-powered artificial muscles require bulky air sources and often lack crucial actuation properties [18]; cables are widely utilized as actuation mechanisms for soft exoskeletons due to their high stiffness. For example, some wearable soft exoskeletons (i.e., exosuits) exploit ribbons [19] or Bowden cables to transmit tensional forces and generate assistive torques on the joints; thus, they can act much like real human muscles. Exosuits can provide effective assistance for hip extension [19, 20], hip flexion, and ankle plantar flexion during toe-off phase [20–22] with large savings of energy during walking. A person walking on a treadmill at 1.5 m/s, carrying a load equal 30% of his weight, can reduce his metabolic rate by 15% if he wears an exosuit [23].

**Figure 1:**
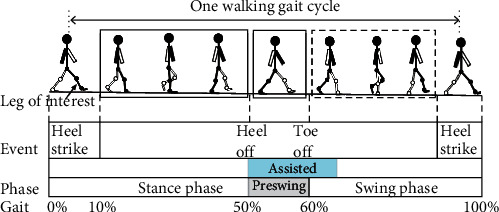
Illustration of the assistance provided by a lower-limb soft exoskeleton during a walking gait cycle in which assistive forces were supplied for hip flexion during the preswing phase.

In a single gait cycle, the leg that swings backward relative to the body can be called the “stance leg” and the leg that swings forward can be called the “swing leg.” Most exosuits provide assistance during the various phases of the stance leg and the toe-off phase of the swing leg (Figure 1). However, the other phases of the swing leg (e.g., the preswing phase) are often neglected. Analyses of these phases are crucial for patients who lack muscle strength in their lower limbs. Therefore, the aim of this study was to design a soft exoskeleton to provide hip flexion assistance during walking for wearers who can walk but lack either leg strength or stamina. The soft exoskeleton should be lightweight and highly compliant with human bodies to avoid imposing kinematic constraints on wearers conducting daily activities. Cables with spools are commonly used as actuators for soft exoskeletons because they are compact and require little space. However, because force transmission relies on the frictions between the spools and cables, slip problems occur and cause drifts in the angular positioning of gaits. Additional space is required for installing force sensors when cables are slacked. In this study, a single-motor-based actuation mechanism was designed; this mechanism enabled the proposed lightweight exoskeleton and prevented the potential slip problems of rotary motors. The actuation mechanism was based on a pulley system that converted the power supplied by the single motor into linear, reciprocating slider motions. When the single motor rotated, the slider moved linearly in one direction to pull the appropriate knee brace with an assistive force of 100 N through cables.

This paper is organized as follows.  Section 2 introduces the system overview of the soft exoskeleton and its actuation mechanism.  Section 3 presents kinematic analyses and simulations to discuss the conservation of mechanical power in the soft exoskeleton. Sections 4 and 5 describe the designs and control strategy of the soft exoskeleton, respectively. Finally, Section 6 reports how the soft exoskeleton was experimentally examined with several subjects to investigate the metabolic costs and the influences on the gaits. The crucial results and conclusions are summarized in Section 7.

## 2. System Overview

**Figure 2:**
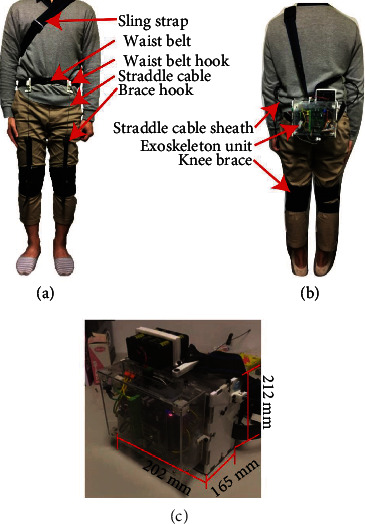
(a) Front and (b) back views of the wearable soft exoskeleton. (c) Exoskeleton system comprising a start button, an actuation unit, sensors, a controller, and a battery.

Figures 2(a) and 2(b) display the proposed lower-limb soft exoskeleton comprising a sling strap, a waist belt, knee braces, straddle cables, and an exoskeleton unit. This device provides assistive forces to the human body during walking by pulling the straddle cables connected to knee braces for hip flexion assistance. The length, width, and height of the exoskeleton unit are 202, 165, and 212 mm, respectively. The exoskeleton unit comprises a start button, an actuation unit, sensors, a controller, and a battery (Figure 2(c)). The exoskeleton unit weighs 4.3 kg and contains a motor that can provide a 100 N force at either knee brace.

**Figure 3:**
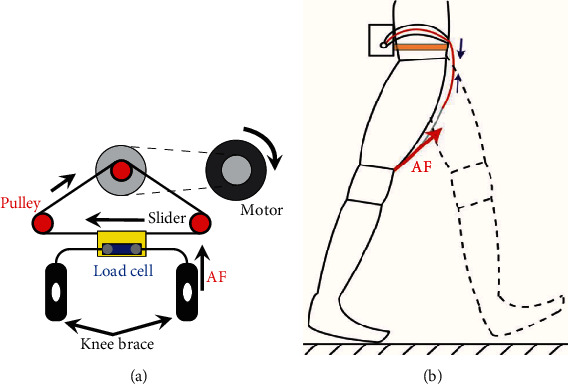
Illustration of the actuation mechanism. (a) When the motor rotates, the slider moves through the pulley system and provides assistive forces for hip flexion by pulling the straddle cables of the knee braces and slacking the straddle cable of the other knee brace. The motor rotates reversely for providing hip flexion assistance to the other leg. (b) During the preswing phase, the assistive force is applied at one knee brace because the motor pulls a straddle cable.

Figure 3 displays the actuation mechanism of the soft exoskeleton. The motor rotates clockwise and moves the slider to the left based on the pulley system displayed in Figure 3(a) when the actuation is triggered at the preswing phase of the right leg. The linear motions of the slider pull the knee brace of the right leg by the straddle cable and convert the motor rotation into assistive force applied at the right knee brace. In this case, the straddle cable of the left knee brace is slacked. For the next gait cycle, the motor must rotate in a counter-clockwise manner to assist hip flexion for the left leg by pulling the straddle cable of the left knee brace. During either preswing phase, the assistive force is applied at one knee brace because the system pulls the straddle cable connected to the knee brace, as displayed in Figure 3(b).

## 3. Modeling and Simulations

### 3.1. Two-Dimensional Kinematic Model of the Human Legs

**Figure 4:**
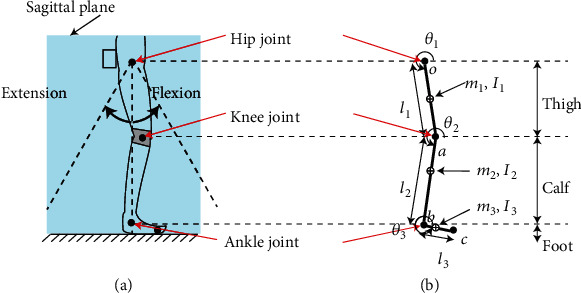
Two-dimensional kinematic model of a human leg.

A two-dimensional (2D) kinematic model of a human leg (Figure 4) was used to analyze the mechanical power required for hip flexion or extension during a walking gait cycle. The thigh, calf, and foot were modeled as linkages joint at the ends, and their rotation was limited to the sagittal plane. In Figure 4, *o*, *a*, *b*, and *c* represent the hip joint, knee joint, ankle joint, and toe, respectively. The thigh, calf, and foot lengths were *l*_1_, *l*_2_, and *l*_3_, respectively. The central masses of the thigh, calf, and foot were denoted as *m*_1_, *m*_2_, and *m*_3_, respectively, located on 44% of *l*_1_ from *o*, 40% of *l*_2_ from *a*, and 25% of *l*_3_ from *b*, respectively. The moments of inertia at the central masses of the thigh, calf, and foot were *I*_1_, *I*_2_, and *I*_3_, respectively. The rotation angles of the thigh, calf, and foot linkages were *θ*_1_, *θ*_2_, and *θ*_3_ with respect to the horizontal ground. The physical parameters of the 2D model of the human leg are summarized in Table 1 [24]. Based on the average of the ground reaction force and gait locomotion data obtained from 20 young people and 20 adults reported in [25], the mechanical power required for hip flexion and extension could be obtained by multiplying the hip rotation moments and the angular velocity of the walking gait cycle through the well-known inverse dynamic method. The simulation results are described later.

### 3.2. Modeling of the Soft Exoskeleton

**Figure 5:**
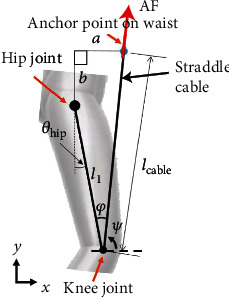
Two-dimensional modeling of a human leg wearing the soft exoskeleton in which the assistive forces were applied at the knee joint by pulling the straddle cable. AF: assistive forces.

Figure 5 displays a 2D model of a human leg wearing the proposed soft exoskeleton in which assistive forces are supplied at the knee braces through the straddle cables. To study the effects of wearing the soft exoskeleton on the mechanical power required for hip flexion and extension, the assistive forces were included in the aforementioned inverse dynamic analysis.

According to the 2D geometric model, the anchor point on the waist had a relative horizontal distance of *a* and a vertical distance of *b*. The straddle cable with a length of *l*_cable_ was connected the anchor and knee points for assisting hip rotations. The convectional hip rotation angle *θ*_hip_ is the angle between the thigh and the vertical axis and can be expressed as follows:

*θ*_hip_ = *θ*_1_ − 270°. Here, *φ* represents the angle between the tight and the straddle cable and *ψ* is the angle between the straddle cable and horizontal axis. The orientation of the straddle cable varied with the hip rotation during walking, thus indicating that *l*_cable_ is a function of *θ*_hip_ and can be expressed as follows:

(1) $$\begin{array}{c} l_{\text{cable}}=\sqrt{a^{2}+b^{2}+l_{1}^{2}-2l_{1}\sqrt{a^{2}+b^{2}}\times \cos\left(\pi -\tan^{-1}\left(\frac{a}{b}\right)-\theta_{\text{hip}}\right).} \end{array}$$

With variation in the orientation of the straddle cable, the angle *φ* varied as the hip rotated and can be expressed as follows:

(2) $$\begin{array}{c} \varphi =\cos^{-1}\left(\frac{l_{\text{cable}}^{2}+l_{1}^{2}-\left(a^{2}+b^{2}\right)}{2l_{1}l_{\text{cable}}}\right). \end{array}$$

Because the direction of the assistive force was in parallel with the straddle cable, the horizontal and vertical components of the assistive force could be obtained based on *φ* as follows:

(3) $$\begin{array}{c} \psi =\frac{\pi }{2}-\left(\varphi -\theta_{\text{hip}}\right) \end{array}$$

In the context of the assistive forces in the inverse dynamic analysis, the mechanical power required for hip flexion and extension while wearing the soft exoskeleton is described in the next section.

### 3.3. Simulation Result

**Figure 6:**
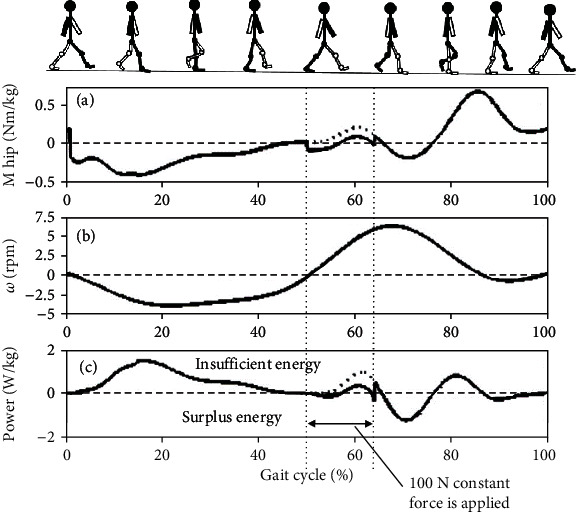
Simulated (a) hip moment, (b) angular velocity of the hip joint, and (c) mechanical power during a walking gait cycle. Here, the solid and dotted lines represent the cases in which the soft exoskeleton is not worn and worn, respectively. A constant assistive force was applied during the preswing phase for conserving power.

To investigate the influence of the soft exoskeleton on energy conservation, the mechanical power values obtained when the soft exoskeleton was worn and not worn were obtained through the inverse dynamic method. We assumed that our device could provide a constant assistive force of 100 N for hip flexion assistance during the preswing phase for 50% to 65% of a gait cycle. As mentioned in the previous section, the direction of the assistive force varied as with the angular position of the hip (Equation (1)).  Figure 6 presents the simulated hip moment, angular velocity of the hip joint, and mechanical power obtained during a walking gait cycle. Analytical simulations were conducted using MATLAB (MathWorks, Massachusetts, United States). The solid and dotted lines represent the cases in which the soft exoskeleton is worn and not worn. In Figure 6(c), the positive value of power indicates the required mechanical power supplied by humans for normal gait motions. In other words, the positive area represents the energy consumed by humans while walking.  Figure 6(a) displays that the magnitude of hip moment required during the preswing phase decreased due to the assistive force provided by the soft exoskeleton. This result indicated that less hip moment was required for swinging the leg forward. Moreover, the mechanical power decreased during the preswing phase, thus decreasing the energy required from 0.3440 to 0.2873 J/kg per cycle. The assistive force provided by the soft exoskeleton could provide hip flexion assistance by conserving 3.589 J of energy required during walking.

## 4. Designs and Assembly of Soft Exoskeleton

### 4.1. Pulley System

**Figure 7:**
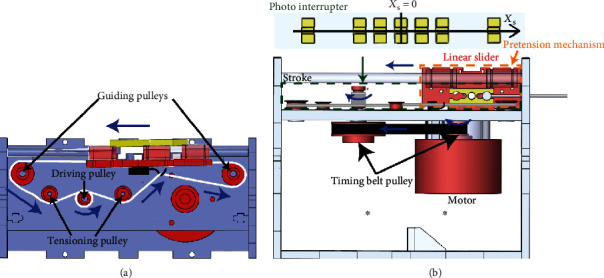
Illustration of the power transmission based on the slider and the pulley system in the (a) top and (b) side views.

Most cable-driven actuations of the soft skeleton tend to exhibit slip problems. Deviations in the hip rotation positions were observed due to these problems. To avoid these problems, a slider with reversible linear motions actuated through the pulley system was developed in this study (Figure 7). All rotating components were installed on an 8 mm thick acrylic board base (Figure 7(a)). A brushless DC motor and two timing belt pulleys were mounted on the rear side of the base, and four pulleys, including guiding and driving pulleys, were mounted on the front side of the base. The two timing belt pulleys formed a gear train with the gear ratio of 3 to drive the pulley for scrolling a nylon thread whose both ends were attached to the two sides of the slider (Figure 7(b)). When the motor rotated, the timing belt pulley pulled the nylon thread and actuated the slider to move in one direction through the driving and guiding pulleys. Because the pulley system was operated through the nylon thread and the end of the nylon thread was not fixed on the driving pulley, friction forces between the nylon thread and the driving pulley were key factors that caused slip problems at the nylon thread. To prevent these slip problems, the driving pulley was wrapped with eight turns of nylon thread. The number of turns was limited because a high number of turns of the threads could increase the frictional forces of the rotating pulleys. Without self-locking effects and a relative low gear ratio of the timing belt pulley, reversible motions of the slider were achieved. An advantage of the linear slider was that the positioning drift could be eliminated by tracking the positions of the linear sliders by photointerrupters, as described later.

### 4.2. Pretension Mechanism

**Figure 8:**
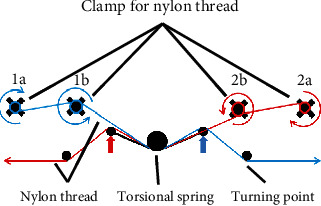
Diagram of the pretension mechanism and fixation of nylon threads mounted on the slider.

In addition to the friction coefficients and wrapping turns, the tensions in the nylon threads served a crucial role in precisely positioning the slider. Because the nylon thread exhibited a large tensional stiffness value, small assembly misalignments or structure deformations could easily slack the nylon thread. Although the thread had been wrapped multiple times, slips occurred when the nylon thread slacked. Therefore, a pretension mechanism was used to maintain the tightness of the nylon threads and was mounted on the slider (Figure 7(b)). To simplify the assembly, a torsional spring was incorporated in the slider with two ends of the spring tangled with each of its arms (Figure 8). The spring was pinned on the slider at its center. The spring could rotate with a small angle such that a 1 mm position deviation of the slider was allowed. The spring was twisted when the slider was loaded, thus allowing another 1 mm position deviation. Two screws and nuts were used for each end of the nylon thread to fix both ends firmly, which are marked as 1a, 1b, 2a, and 2b in Figure 8. Before the nylon thread was tightened, the nylon thread was wrapped with one turn on each screw in a clockwise manner on 1a and 2a and in a counter-clockwise manner on 1b and 2b. Then, the nuts on 1a and 2a were screwed to hold and tighten the nylon thread. Finally, the nuts were screwed on 1b and 2b to fix the nylon thread. As the nylon thread undergoes tension, the friction force between the nuts and the nylon thread tightens the nut further due to the screw and nut mechanism. Thus, the clamp of the nylon thread does not loosen, even after prolonged stress.

### 4.3. Anchors and Connectors

We used straddle cables to transmit assistive forces from our device to the knee braces and then to the legs of the exosuit wearer. The connections between the cable and the knee braces were designed to be easily adjustable for different wearers. Because the straddle cables lacked flexibility and adjustability and could not be tied with a knot on the knee braces, Velcro was used to adhere the straddle cables to the knee braces for ensuring reliable attachments.

**Figure 9:**
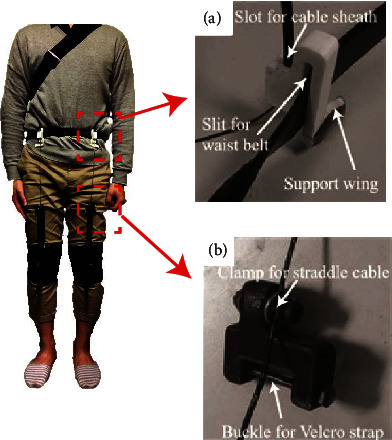
(a) Anchors and (b) connectors mounted on the waist.

Figure 9(a) shows the anchor of the soft exoskeleton composed of the slot for holding the cable sheath and the open slit for attachment to the waist belt. Since the anchor attached to the waist close to the hip, walking might cause discomfort to the pelvic region; to maximize comfort, the 3D-printed anchor had a support wing filled with foam that distributed pressure on the hip. Figure 9(a) shows the 3D-printed connector composed of an M4 screw, an M4 nut, and spacers. By threading the straddle cables through the spacers and clamping it with the screw and the nut, we fixed the 3D-printed connector tightly with the straddle cables. Then, the Velcro strap on the knee brace was wound with a buckle to ensure that the Velcro strap would remain fastened during walking. The rest of the strap was wound on the 3D-printed connectors.

## 5. Sensors and Control Strategy

### 5.1. Load Cells

**Figure 10:**
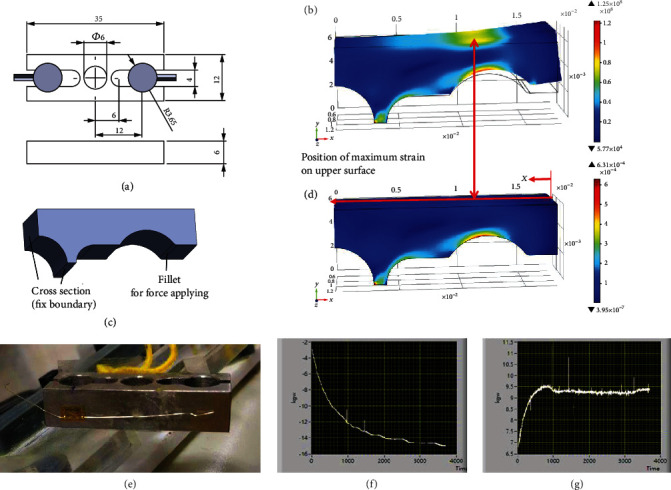
FEM simulations of the load cell. (a) Geometrical dimension (unit: mm). (b) Simplified quartered model. (c) Principal stress distributions. (d) Principal strain distributions. (e) Photograph of the load cell. (f, g) Drift effects of the left and right sides of the load cell.

Instead of using commercial load cells, a compact twin pull-type load cell was designed for the soft exoskeleton, as illustrated in Figure 10. In Figure 10(a), the load cell connected the heads of the straddle cables and the slider for pulling the knee braces; because the load cell had been designed to be compact, it only occupied a small volume. Finite element simulations were conducted for the designs of the load cell in Comsol commercial software. The simplified quartered model based on two symmetric surfaces is presented in Figure 10(b). Because the maximum assistive force was 100 N for the soft exoskeleton, the load cell was designed to measure the maximum assistive force of 200 N with a safety factor of 2. When a straddle cable applied a tensile assistive force of 200 N in the *x*-direction, the force was applied at the contact point with the *x*-component of 100 N and *y*-component of 65.5 N due to the contact angle of 33.23° between the load cell and head of the straddle cable. We assumed the force was exerted on the fillet surface with a radius of 0.5 mm, which was the surface between the head of the straddle cables and load cells. The load cell was made of stainless steel; it was designed to endure a maximum yield stress no greater than 200 MPa. The simulation results are shown in Figures 10(c) and 10(d).

The maximum stress was 125 MPa and was 37.5% smaller than maximum yield stress, thus indicating that the load cell could withstand a force of 20 kgw. The maximum strain occurred on the upper surface of the load cell at a distance of a 14 mm from the center of the load cell in the longitudinal direction. This phenomenon caused a small elongation of 2.04 *μ*m, and a fine resolution could not be attained for the general metallic foil-type strain gages. Therefore, strain gages with a large gage factor (GF) as that of semiconductor-type strain gages should be used for our device. Multiple strain gages (Kyowa: KSN-2-120-E3-16) with a GF of −105 were implemented using two quarter-bridge Wheatstone bridges for the twin load cell. The load cell was fabricated and calibrated experimentally at a measurement range of 0 N–100 N and a resolution of 1.76 N (Figure 10(e)). Although the load cell exhibited a fine resolution and good linearity in the force measurements, the drift effect due to the strain gages and the increase in the temperature of the circuit were not negligible. Figures 10(f) and 10(g) display the drift effects of the left and right sides of the load cell, in which the measured force varied and saturated as time increased. Therefore, the measured data of load cells were used as trigger signals in our preliminary work because accurate values of the assistive forces could not be provided.

### 5.2. Coordinate Mapping

Because the actuation mechanism converted the motor rotations to the linear motions of the slider, the coordinates of the hip angular positions were mapped to linear positions set on the sliders. The hip rotation angles were in the range of −10° to 10° during the preswing phase. Moreover, a linear relation existed between the length of cable *l*_cable_ and the hip rotation angle *θ*_hip_, which is insensitive to the changes in the geometric parameters of *l*_1_, *a*, and *b*, (Figure 4). Equation (1) simplified the linearization method with a small variation in the hip rotation angle:

(4) $$\begin{array}{c} l_{\text{cable}}\approx 0.1732\theta_{\text{hip}}+c_{1,} \end{array}$$

where *c*_1_ is a constant based on the thigh length. By defining *c*_1_ = *l*_cable_ at *θ*_hip_ = 0, the mapped coordinate of the slider *X*_s_ was defined as the origin at which *X*_s_ = 0 cm when the slider was at the midpoint of the stroke at length of 90 mm (Figure 7(b)). Based on the above condition, the formula of *X*_s_ can be presented as follows:

(5) $$\begin{array}{c} X_{\text{s}}=0.1732\theta_{\text{hip}.} \end{array}$$

**Figure 11:**
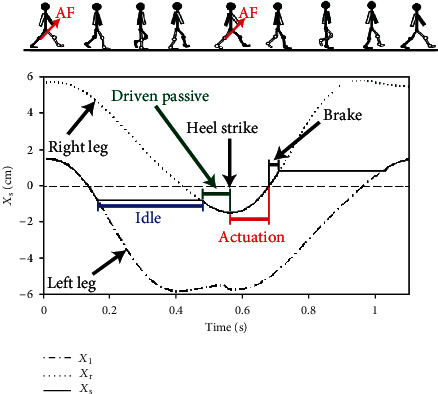
Illustrations of the motion projections of the mapped coordinate for the cases in which the soft exoskeleton is worn and not worn while walking. The assistive forces were provided during the preswing phases for each leg.

The thigh motions were projected to the mapped coordinates by substituting *θ*_hip_ into Equation (5). Here, the targeted hip extension or flexion angles in the range of −10° to 10° were mapped to the *X*_s_ range of −2 to 2 cm for the mapped coordinates. We assumed that the walking gait of a normal individual was bilaterally symmetrical in terms of the phase and motion. Therefore, the slider motion projection of the left thigh *X*_l_ was a mirrored version of the right thigh *X*_r_ with a 50% phase lag during gait cycle. The slider motion projections for both thighs when the soft exoskeleton was not worn are displayed in Figure 11. In this figure, the dotted line represents the right leg and the dotted–dashed line represents the left leg.

### 5.3. Control Strategy

**Figure 12:**
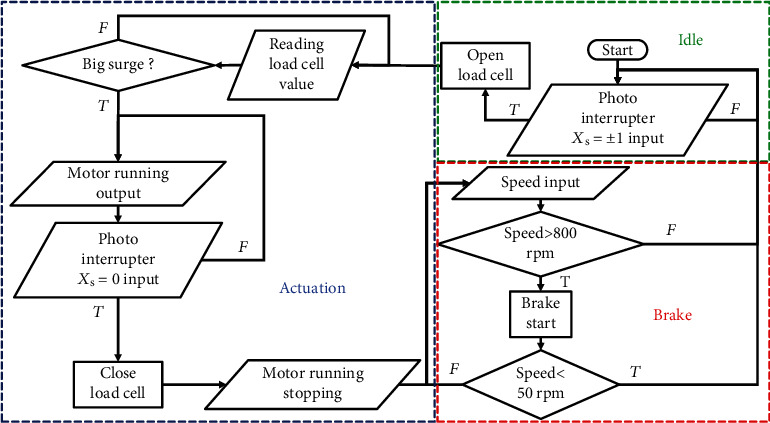
Flowchart of the control algorithm.

A position-based control strategy was used for the linear actuation mechanism in this study. Seven photointerrupters were mounted along the stroke with the mapped positions of *X*_s_ = −4.5, −2, −1, 0, 1, 2, and 4.5 cm to track the positions of the linear motions of the slider.  Figure 12 displays the flowchart of the control strategy that was realized for different stages—idle, wearer-driven pulling, actuation, and brake stages. They were determined using the motor rotational speed measured from the embedded speed sensor of the motor. If the motor rotation was higher than 800 rpm, then brakes were applied automatically and decelerated until the rotation speed was lower than 50 rpm. The motor rotational speed was zero while walking. Moreover, the soft exoskeleton was in the idle stage, and the slider was at the position of *X*_s_ = −1 or 1 cm at which the straddle cables of both legs were slacked. The load cells were enabled to monitor the tensile forces of the straddle cables connected to the knee braces. During the end period of the backward swinging of legs, the slider was moved away from the positions of *X*_s_ = ±1 cm and the soft exoskeleton entered the wearer-driven pulling mode. At the beginning of the preswing phase, one side of the load cell detected a high decrease in the measured force when the leg swung forward. The actuation mechanism was triggered when the motor begin to rotate and thus the slider began to move linearly to provide hip flexion assistance; this period can be known as the actuation period. When the slider passed the position of *X*_s_ = 0 cm, the soft exoskeleton entered the brake stage and the motor rotated in the opposite direction to decelerate the linear motion of the slider until the slider stopped at the position of *X*_s_ = ±1 cm. Then, the slider remained at the position of *X*_s_ = ±1 cm and the soft exoskeleton entered the idle stage until the next actuations. The photointerrupters at *X*_s_ = −4.5, −2, 2, and 4.5 cm were designed to ensure that the slider returns to the position of *X*_s_ = 0 cm in our original design, which is not conducted in the present study.

### 5.4. Actuation Trajectory

The motion trajectory of the linear slider while wearing the soft exoskeleton is shown in Figure 11 based on the control strategy. At the beginning of the preswing phase, that is, after the heel of the right leg strikes the ground (*t* = 0 s), the actuation mechanism was activated due to a rapid decrease in the force measured force using the load cell and an assistive force was provided at the right knee by pulling the straddle cable through the linear movement of the slider by the counter-clockwise rotation of the motor. When the slider passed the position of *X*_s_ = 0 cm, the soft exoskeleton entered the brake stage and the motor rotated in a clockwise manner to decelerate the slider until the slider stopped at the position of *X*_s_ = −1 cm. Subsequently, the soft exoskeleton entered the idle stage and the slider remained at the same position of *X*_s_ = −1 cm at which the straddle cables of both legs were slacked. Similarly, at the end period of the backward swinging of the left leg, the slider was pulled passively and moved away from the position of *X*_s_ = −1 cm. Then, at the beginning of the preswing phase of the left leg, the actuation mechanism was triggered and the motor rotated in a clockwise manner to provide an assistive force at the left knee for hip flexion assistance. At this point, the slider moved to the position of *X*_s_ = 1 cm due to the motor and pulled the straddle cable connected to the left knee. When the slider passed the position of *X*_s_ = 0 cm, the soft exoskeleton entered the brake stage and the motor rotated in a counter-clockwise manner to decelerate the slider until the slider reached the position of *X*_s_ = 1 cm. Then, the slider remained at the position of *X*_s_ = 1 cm and the soft exoskeleton entered the idle stage until next gait cycles.

## 6. Experimental Results and Discussions

### 6.1. Metabolic Energy Conservation

**Figure 13:**
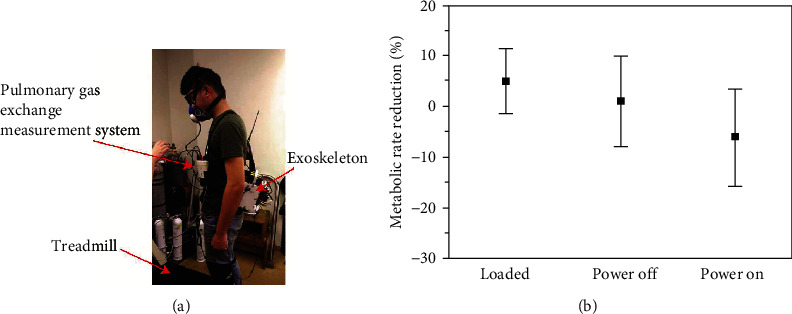
(a) Photograph of the metabolic tests conducted while walking on a treadmill. (b) The measured statistical distributions of the metabolic rate reduction for the cases in which loads are carried, power is switched off, and power is switched on. The error bar indicated one standard deviation. The metabolic rates were normalized to the case in which only the knee braces were worn (i.e., without wearing the soft exoskeleton).

To evaluate the capabilities of conserving energy when a wearer of the soft exoskeleton walks, metabolic tests were conducted using a treadmill machine (Figure 13(a)). Seven participants with ages ranging from 23 to 24 years, height ranging from 171 to 180 cm (average = 174 cm, standard deviation (SD) = 3.16 cm), and weight ranging from 67 to 72 kg (average = 70 kg, SD = 1.91 kg) participated in the tests. The treadmill was setup at a constant walking speed of 4.3 km/h. The metabolic rates of the subjects were measured and recorded using a portable pulmonary gas exchange measurement system (K4b2, COSMED s.r.l., Rome, Italy) during the test. The tests included four scenarios: (a) wearing the knee braces only (i.e., free walking without wearing the soft exoskeleton), (b) wearing the soft exoskeleton when the straddle cables were loosened (i.e., carrying loads), (c) wearing the soft exoskeleton when it is switched off, and (d) wearing the soft exoskeleton when it is switched on. For the scenarios (c) and (d), a 2 kgw pretensioned force was applied while wearing the soft exoskeleton. Each test lasted for 7 min. Because our device was a prototype that had not been optimized for weight and comfort, we focused on the effects of the assistance and ignored the influence of carrying loads. To ensure that the soft exoskeleton could be triggered appropriately and could actuate correctly, the straddle cables were pretensioned with a 2 kgw force while a subject was wearing the soft exoskeleton. Moreover, the anchors on the waist were adjusted such that the cables were in parallel with the sagittal plane.

Figure 13(b) displays the measured statistical distributions of the metabolic rate reduction for the cases in which the soft exoskeleton was worn with loosened straddle cables, worn when it is switched off, and worn when it is switched on. The metabolic rate reduction is expressed as follows:

(6) $$\begin{array}{c} \;\frac{{MR}_{\text{m}}-{MR}_{\text{f}}}{{MR}_{\text{f}}}. \end{array}$$

Here, *MR*_m_ is the metabolic rate when the soft exoskeleton is worn for three cases and *MR*_f_ is the metabolic rate obtained without wearing the soft exoskeleton. The negative values represented that energy was conserved due to the assistance provided by the exoskeleton, and the positive values presented that energy consumption occurred when loads were carried. These results demonstrated that the metabolic rate could be reduced further due to the assistive forces provided by our device while walking, thus indicating that the proposed soft exoskeleton can conserve energy when the wearer walks. The deviations were due to the unfamiliarity in using the exoskeleton or lack of soft exoskeleton training.

### 6.2. Impacts on Gaits

**Figure 14:**
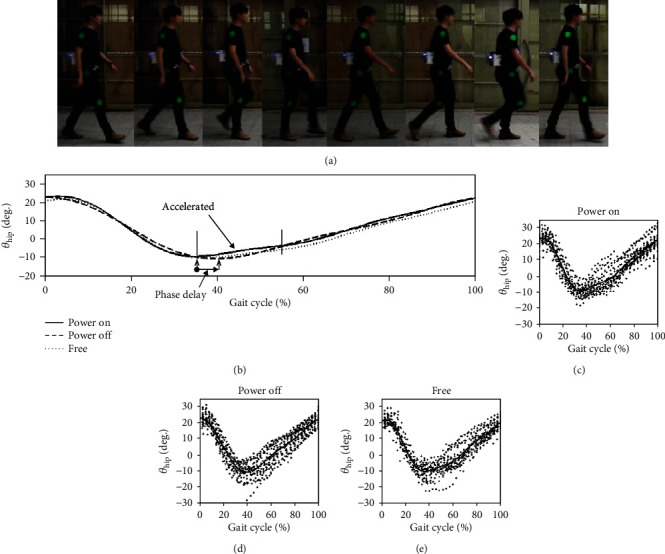
(a) Photograph of the gait motion measured using the video camera. (b) Averaged gait motions for the cases in which the soft exoskeleton is worn with its power switched on, is worn with its power switched off, and is not worn. An assistive force was provided by the soft exoskeleton during the preswing phase. (c, d, e)All gait motions for each scenario.

To investigate the effects on gait motions when the soft exoskeleton is worn, gait measurements were conducted for the same subjects. Subjects wore a soft exoskeleton and walked along a straight line with three markers attached at their shoulders, hips, and knees. Moreover, the angular rotation positions of the hip and knees were recorded using a video camera located at a distance of 5.3 m in front of the straight line (Figure 14(a)). The experiments were conducted for the participants under three scenarios—wearing the soft exoskeleton when it is switched on, wearing the soft exoskeleton when it is switched off, and wearing knee braces only (i.e., free walking without wearing the soft exoskeleton). In the first two scenarios, a 2 kgw pretensioned force was applied while when the soft exoskeleton was worn. The gait patterns were recorded at a frame rate of 30 fps. Each frame was analyzed with image processing techniques to track the positions of three markers. The angle formed by the markers were measured, transformed, detrended, and finally separated through every two heel strikes in one gait cycle.

Figure 14 displays the measured gait motions of the subjects. The solid lines represent that the soft exoskeleton was worn and was powered on, and the dashed lines represent that the soft exoskeleton was worn and was powered off. The dotted lines represent that only the knee braces were worn (i.e., free walking). The three curves presented in Figure 14(b) were obtained through fitting the curves of all the gait motions for the scenarios presented in Figures 14(c)–14(e). A phase lag from 35% to 56% is displayed in Figure 14(b); subjects attain the maximum hip extension while wearing the device when it is switched off relative to the free walking case. This behavior was observed because that the slider remained at the last position after pulling and could not return to the central point when the device was switched off. When the soft exoskeleton was switched on, the damping effect was minimized because the slider could move and return to the central position after being pulled by the subject. Therefore, the phase lag of the maximum hip extension was eliminated when the soft exoskeleton was switched. Moreover, hip rotation accelerated in the preswing phase from 35% to 56% due to the hip flexion assistance provided by the soft exoskeleton (Figure 14(b)). Because no obvious changes were observed in the gait, the proposed soft exoskeleton is concluded to have at most a slight influence on the gait motions.

## 7. Conclusions and Future Work

A compact lower-limb soft exoskeleton was presented in this study for providing hip flexion assistance to walking wearers with low muscle strength. A lightweight exoskeleton was designed to minimize slip problems; the soft exoskeleton was designed to actuate both legs through a single actuator by converting the motor rotations into linear reciprocating motions of the slider through a pulley system. According to the simulation results, the exoskeleton was capable of conserving the energy required during the preswing phase of a gait cycle. A prototype was fabricated, assembled, and experimentally examined through metabolic rate tests. The results revealed that our device could reduce the metabolic cost of walking and exerted at most a slight influence on gait motions, thus indicating that the proposed soft exoskeleton could conserve energy during hip flexions.

Some future studies are under investigation. Because the actuation speed of straddle cables is limited, the current actuation mechanism cannot be used to attain a high walking speed. Moreover, the load cell has a significant drift effect because of the temperature rise in the strain gage and circuitry. This problem can be overcome by finely soldered circuitry and temperature compensations. Moreover, the measured load information can be utilized for feedback control. Because the soft exoskeleton was designed to provide assistance for the preswing phase of a gait cycle, our device could be integrated with other exosuits to provide assistance for entire gait cycles in the future. The proposed single actuator-based actuation mechanism is expected to be beneficial for realizing a compact and lightweight soft exoskeleton.

## Figures and Tables

**Table 1:** Physical parameters of the human legs [23].

Thigh		Calf		Foot	
*l*_1_	41 cm	*l*_2_	41 cm	*l*_3_	20 cm
*m*_1_	7.7 kg	*m*_2_	3.1 kg	*m*_3_	0.8 kg
*I*_1_	1093 kg/cm^2^	*I*_2_	406 kg/cm^2^	*I*_3_	31 kg/cm^2^
CM to “*o*”	44% of *l*_1_	CM to “*a*”	40% of *l*_2_	CM to “*b*”	25% of *l*_3_

## Data Availability

The measured data used to support the findings of this study are available from the corresponding author upon request.
